# The Association Between Maternal ABO Blood Group and the Occurrence of Spontaneous Preterm Birth: A Retrospective Population-Based Cohort Study

**DOI:** 10.1007/s43032-024-01705-6

**Published:** 2024-09-28

**Authors:** Eyal Rom, Manal Massalha, Offer Erez, Raed Salim

**Affiliations:** 1 Department of Obstetrics and Gynecology, Emek Medical Center, Afula, Israel; 2 Rappaport Faculty of Medicine, Technion, Haifa, Israel; 3 Department of Obstetrics and Gynecology, Soroka University Medical Center, Beer Sheva, Israel; 4 Department of Obstetrics and Gynecology, Wayne State University School of Medicine, Detroit, MI USA; 5 Department of Obstetrics and Gynecology, Holy Family Hospital, Nazareth, 16100 Israel; 6 Azrieli Faculty of Medicine, Bar-Ilan University, Safed, Israel

**Keywords:** ABO blood group, Adverse pregnancy outcome, Placental abruption, Preterm birth, Preterm rupture of membranes

## Abstract

There is limited and inconsistent evidence that imply a relationship between ABO blood types and rate of preterm birth (PTB). We aim to examine the association between maternal ABO blood group and PTB rate. A retrospective-study conducted at a university teaching institution on data collected between 2013 and 2019. Women who delivered a viable neonate at ≥ 24 weeks without major malformations were included. Indicated PTBs were excluded. PTB and early PTB were defined as deliveries that occurred < 37 and < 34 weeks respectively. PTB was further divided into 3 subgroups according to etiology: membranes rupture, intact membranes, and placental abruption regardless of membranes’ status. The primary outcome was spontaneous PTB rate. Of 19,301 women included, PTB and early PTB rates were 7.3% (1,418/19,301) and 2.3% (440/19,301) respectively. Rates of PTB in blood groups A, B, O, and AB, were 7.3%, 6.9%, 7.5%, and 7.5% respectively (*p* = 0.68). There was no significant difference according to etiology. Rates of early PTB were also comparable (*p* = 0.63). After adjustment for demographic and obstetric variables, blood type was associated with increased placental abruption rate among women who had early PTB (*p* = 0.038). Placental abruption rate was significantly higher in group A (22.5%) compared to group B (14.1%), (adjusted *p* = 0.04) and group O (14.0%), (adjusted *p* = 0.01). The rate in group AB was 17.1%, (adjusted *p* = 0.85). In conclusion, no association was found between a particular blood group and PTB rate. Women with group A, admitted in early PTB, had an increased risk that the underlying etiology was placental abruption.

## Introduction

Pre-Term Birth (PTB) is responsible for nearly one million newborn fatalities and is the greatest cause of death in children under the age of five years [1]. Worldwide, an estimated 13.5 million neonates are delivered prematurely. Around 65% of PTB in 2020 occurred in sub-Saharan Africa and southern Asia, with rates ranging from roughly 4% in Europe to 16% in Africa and southern Asia [2]. Comparable gap was also observed in the United States in 2022. The rate of PTB among Black women (14.6%) was substantially higher than White (9.4%) or Hispanic women (10.1%) [1, 2],.

PTB is a syndrome associated with multiple underlying mechanisms such as intra-amniotic infection/inflammation, accelerated thrombin production and placental vascular dysfunction [3–5]. Preventing PTB requires an understanding of the risk factors and mechanisms of disease that lead to this obstetric syndrome. A previous history of PTB is the most recognized risk factor for PTB. The risk increases after one PTB by 1.5 to 2-fold, and the increase is maximum after several PTB’s [4]. Other conditions that are linked to an elevated risk of PTB include among others short cervix in the index pregnancy, multiple gestations, polyhydramnios, maternal smoking, low body mass index, and pregnancies conceived through assisted reproduction [3, 4].

The association between spontaneous PTB and maternal blood type is not completely understood. The existed literature points to a possible expression of pro-inflammatory mediators or to particular placental factors, that may affect the risk of PTB. The expression of these variations may be detected particularly among women with a certain blood type [6–10]. Additionally, maternal anti-fetal rejection is another mechanism that could explain the observation of an association among spontaneous PTB and maternal-newborn B-A and AB-B pairs [11], similar to the proposed mechanism behind maternal ABO blood group and preeclampsia and gestational diabetes [12, 13].

Currently, the data regarding the association between maternal ABO blood groups and spontaneous PTB are scarce and inconsistent [11, 14–16]. The aim of this study was to determine the association between maternal ABO blood groups and the risk of PTB in a large population-based cohort study. Considering the wide availability and the low costs of maternal blood group testing, detection of a relationship between maternal ABO blood group and spontaneous PTB, as in several other diseases, may be used as an additional means of screening women in early pregnancy. Furthermore, it may shed light or provide additional clues to the mechanism of PTB, if a relationship is detected.

## Materials and Methods

A retrospective population-based cohort study was conducted utilizing data obtained from a single University Teaching Hospital in Afula, Israel, from January 2013 to December 2019.

All pregnant women who delivered at ≥ 24 weeks of gestation were included in the study cohort. The study cohort was divided into term and PTB groups. Among the PTB group, spontaneous PTB only was included in the analysis. Exclusion criteria, in both groups, included women who had indicated PTB that was defined as any induction of labor, or cesarean delivery, with intact membranes between 24 weeks and week 36 and 6 days. Additional exclusion criteria included women who had a fetal demise or termination of pregnancy due to fatal chromosomal or congenital abnormality, and women with missing ABO blood group information. Women, in both groups, who had more than one birth during the study period were included only once to eliminate selection bias. In these cases, the first delivery was selected among the control group, if no PTB was followed. If a subsequent PTB occurred, this woman was included in the study group, and only her PTB was selected for analysis.

Spontaneous PTB was defined as deliveries that occurred at < 37 weeks, and early PTB was defined as deliveries that occurred < 34 weeks. PTB was further subdivided based on clinical symptoms and physical examination upon presentation to PTB that followed preterm rupture of membranes (PROM) whether in labor or not, non-PROM PTB, and placental abruption regardless of membranes` status. Placental abruption was defined when vaginal bleeding was accompanied with uterine contractions in the absence of placenta previa.

Data were retrieved from the delivery ward’s electronic medical files and cross-checked with records obtained from the hospital admission and discharge registry. Maternal demographic and obstetric variables including gestational age at delivery were manually validated and recorded. Potential risk factors for PTB were also recorded including multiple gestations, uterine anomalies, placenta previa, any infectious disease in pregnancy (e.g., urinary tract infection and gastroenteritis), polyhydramnios defined as amniotic fluid index 25 cm or more, and oligohydramnios defined as amniotic fluid index ≤ 5 cm [17]. GBS status (as determined by a positive urine culture or vaginal-rectal swab during the current pregnancy) was also recorded. Additionally maternal ABO blood group and Rh status were also retrieved from the medical registry.

The primary outcome was the association between maternal ABO blood group and rate of spontaneous PTB. Secondary outcomes included the association among maternal ABO blood group system and spontaneous early PTB, PROM, non-PROM, and placental abruption.

### Ethical Approval

The institutional review board at Emek Medical Center, authorized the study (0067-20-EMC) in May 19, 2020. Due to the retrospective nature of the study, signed informed consents were exempted by the ethics committee.

### Statistical Analysis

The demographic and obstetric characteristics of preterm and term women were compared using X^2^ tests or Fisher’s exact test. Odds ratios and 95% confidence intervals were calculated. Logistic regression was used to identify independent risk factors for PTB among the ABO blood groups after controlling for confounding factors such as age, multiple pregnancy, placenta previa, malformed uterus, GBS status, any infectious disease, oligohydramnios, and fetal abnormality. To assess the effect of blood group on gestational age, linear regression was performed. This was repeated after controlling for confounding factors such as maternal age, multiple pregnancy, placenta previa, malformed uterus, GBS status, infectious disease, and abnormal amniotic fluid indexes. A *p* < 0.05 was considered significant. All analyses were performed using SPSS (IBM SPSS Statistics for Windows, Version 24.0. Armonk, NY: IBM).

## Results

**Fig. 1:**
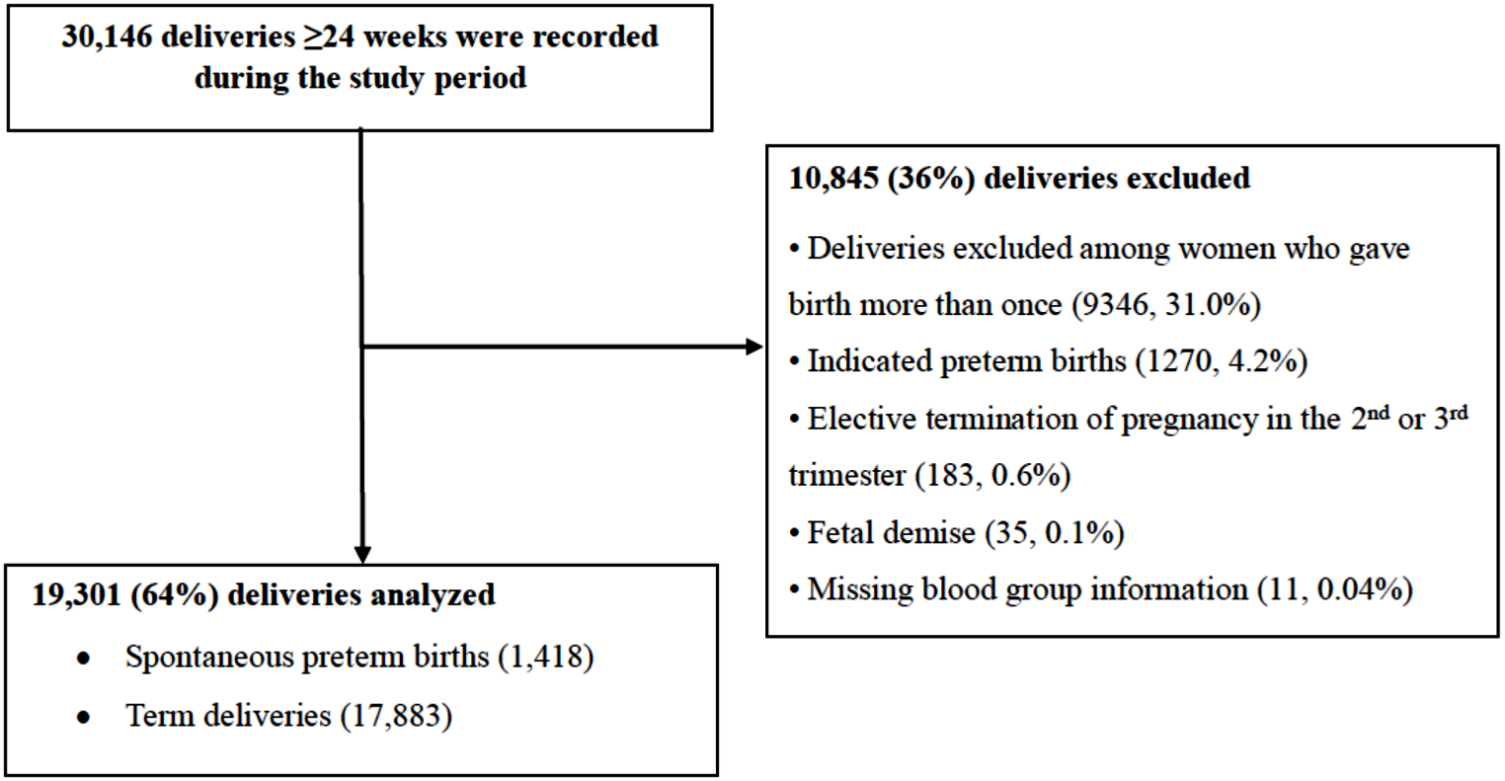
Diagram detailing the excluded deliveries from the cohort

Over all 30,146 deliveries ≥ 24 weeks were recorded during the study period. Among all deliveries, 9346 (31.0%) belonging to women who gave birth more than once, were excluded. Additionally, women who had indicated PTBs (1270, 4.2%), elective termination of pregnancy in the 2nd or 3rd trimester (183, 0.6%), fetal demise (35, 0.1%), and missing blood group information (11, 0.04%), were also excluded. The remaining 19,301 (64%) deliveries were all included in the analysis (Fig. 1).

Blood group A was the most common (7131, 36.9%), followed by O (7039, 36.5%), B (3787, 19.6%) and AB (1344, 7.0%). Rh negative was recorded in 1,734 (9.0%) women. The rate of spontaneous PTB was 7.3% (1418/19301) and the rate of early PTB was 2.3% (440/19301). Of all the 1,418 PTB, 768 (54.2%) were classified as PROM, 516 (36.4%) with intact membranes and 134 (9.4%) were placental abruptions.

Rates of PTB in blood groups A, B, O, and AB, were 7.3%, 6.9%, 7.5%, and 7.5% respectively (*p* = 0.68). The distribution of ABO blood group was equal between term and preterm deliveries (*p* = 0.68). Additionally, there was no significant difference in the rate of PTB between women with Rh negative compared to Rh positive (*p* = 0.38), (Table 1). Mean maternal age was higher among the PTB group compared to the term group (*p* = 0.001). In addition, the PTB group had significantly higher rates of multiple pregnancies (*p* = 0.001), malformed uterus (*p* = 0.001), placenta previa (*p* = 0.001), GBS carrier status (*p* = 0.001), infectious diseases during pregnancy (*p* = 0.001), gestational hypertension (*p* = 0.001), and oligohydramnios (*p* = 0.001) compared to women who delivered at term. The rate of polyhydramnios did not differ between the groups (*p* = 0.12). Delivery mode differed significantly between the groups (*p* = 0.001). The odds of cesarean delivery rate were 2.42 higher among women who had PTB compared to women who delivered at term (35.8% vs. 18.3%, *p* = 0.001) while the rate of vacuum deliveries was significantly lower (1.7% vs. 4.6%, *p* = 0.001), (Table 1).

**Table 1:** Demographic and obstetric characteristics of women with and without preterm birth

	All women (19301)	Preterm (*N* = 1418)	Term (*N* = 17883)	*P*-value	OR (95%CI)
**ABO blood group**				0.68	
A	7131 (36.9)	523 (36.9)	6608 (37.0)	0.99	1.00 (0.89–1.12) 1.00 (reference)
B	3787 (19.6)	261 (18.4)	3526 (19.7)	0.26	0.92 (0.80–1.06) 0.94 (0.80–1.10)
O	7039 (36.5)	532 (37.5)	6507 (36.4)	0.43	1.05 (0.94–1.17) 1.03 (0.91–1.17)
AB	1344 (7.0)	102 (7.2)	1242 (6.9)	0.77	1.03 (0.84–1.27) 1.03 (0.83–1.29)
Rh Positive Rh Negative	17,567 (91.0) 1734 (9.0)	1281 (90.3) 137 (9.7)	16,286 (91.1) 1597 (8.9)	0.38	1.00 (reference) 1.09 (0.90–1.31)
Age, years (Median; IQR)	29.4 ± 5.7 (29; 25–34)	30.2 ± 5.9 (30; 26–34)	29.4 ± 5.7 (29; 25–33)	0.001	1.02 (1.02–1.04)
Multiple gestation	588 (3.0)	275 (19.4)	313 (1.7)	0.001	13.57 (11.42–16.13)
Uterine anomalies	87 (0.5)	16 (1.1)	71 (0.4)	0.001	2.87 (1.67–4.96)
Placenta Previa	267 (1.4)	136 (9.6)	131 (0.7)	0.001	14.44 (11.28–18.47)
GBS carrier	927 (4.8)	99 (7.0)	828 (4.6)	0.001	1.55 (1.25–1.93)
Infectious diseases	683 (3.5)	140 (9.9)	543 (3.0)	0.001	3.51 (2.89–4.27)
Gestational hypertension	602 (3.1)	70 (5.0)	532 (3.0)	0.001	1.70 (1.32–2.20)
Polyhydramnios	408 (2.1)	38 (2.7)	370 (2.1)	0.12	1.31 (0.93–1.84)
Oligohydramnios	686 (3.6)	24 (1.7)	662 (3.7)	0.001	0.45 (0.30–0.68)
**Delivery mode**				0.001	
Vaginal; spontaneous	14,674 (76.0)	882 (62.2)	13,792 (77.1)		1.00 (reference)
Cesarean delivery	3780 (19.6)	507 (35.8)	3273 (18.3)	0.001	2.42 (2.16–2.72)
Vaginal; operative	847 (4.4)	24 (1.7)	823 (4.6)	0.001	0.46 (0.30–0.68)

Data are mean (± SD) or N (%)

OR, odds ratio; CI, confidence interval

Demographic and obstetric characteristics of the study cohort according to the ABO blood group are presented in Table 2. Ethnicity (*p* < 0.001) differed significantly between the groups. Other demographic and obstetric variables were comparable among the different ABO blood groups. Mode of delivery differed also among the groups (*p* = 0.04). The incidence of vacuum deliveries was lowest among women with group O compared with women in the other blood groups (*p* = 0.02). After adjusting for ethnicity, the difference in the incidence of vacuum deliveries remained significantly lower in group O (*p* = 0.03). The incidence of cesarean deliveries was similar between the groups (*p* = 0.26), (Table 2).

**Table 2:** Demographic and obstetric characteristics of patients according to ABO blood group

Demographic data	Group A (*N* = 7131)	Group B (*N* = 3787)	Group O (*N* = 7039)	Group AB (*N* = 1344)	*P*-value
Age (years)	29.5 ± 5.7	29.4 ± 5.7	29.4 ± 5.6	29.5 ± 5.6	0.89
Ethnic group (*n* = 19301) Jewish (*n* = 10702) Arab (*n* = 8599)	3864 (36.1) 3267 (38.0)	2264 (21.2) 1523 (17.7)	3777 (35.3) 3262 (37.9)	797 (7.4) 547 (6.4)	< 0.001
Chronic hypertension	232 (3.3)	101 (2.7)	214 (3.0)	60 (4.5)	0.01
Gestational hypertension	214 (3.0)	115 (3.0)	216 (3.1)	57 (4.2)	0.11
Placenta previa	116 (1.6)	42 (1.1)	91 (1.3)	18 (1.3)	0.13
Uterine anomalies	29 (0.4)	12 (0.3)	43 (0.6)	3 (0.2)	0.08
Multiple gestation	233 (3.3)	106 (2.8)	202 (2.9)	47 (3.5)	0.30
Group B Streptococcus	353 (5.0)	174 (4.6)	335 (4.8)	65 (4.8)	0.87
Infectious diseases	265 (3.7)	126 (3.3)	246 (3.5)	46 (3.4)	0.74
Polyhydramnios	135 (1.9)	85 (2.2)	164 (2.3)	24 (1.8)	0.23
Oligohydramnios	266 (3.7)	123 (3.2)	253 (3.6)	44 (3.3)	0.57
Fetal abnormality	10 (0.1)	7 (0.2)	7 (0.1)	1 (0.1)	0.66
Delivery mode Vaginal; spontaneous Vaginal; operative Cesarean delivery	5371 (75.3) 342 (4.8) 1418 (19.9)	2913 (77.0) 174 (4.6) 700 (18.4)	5375 (76.4) 267 (3.8) 1397 (19.8)	1011 (75.2) 63 (4.7) 270 (20.1)	0.04 0.18 0.02 0.26
Birthweight, gr	3221 ± 527	3212 ± 530	3214 ± 522	3230 ± 551	0.63

Data are mean (± SD) or N (%)

**Fig. 2:**
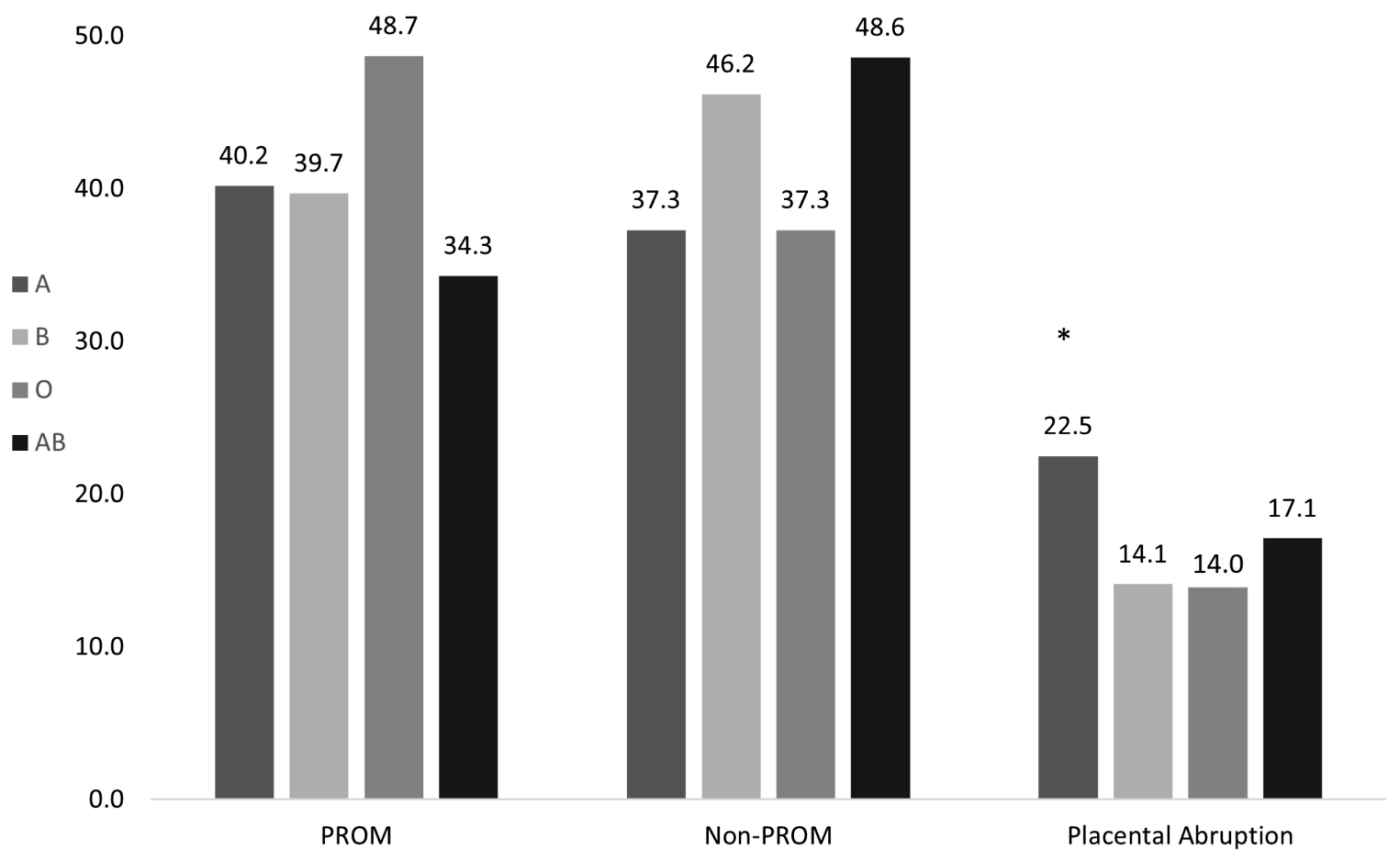
Rates of PTB < 34 weeks among all blood groups according to etiology. PROM, preterm rupture of membranes

Gestational age at delivery and the incidence of PTB among each ABO blood group are presented in Table 3. Mean gestational age at delivery was similar between the groups (*p* = 0.59). After adjusting for maternal age, ethnicity, multiple pregnancies, placenta previa, malformed uterus, GBS status, infectious disease, gestational hypertension, and oligohydramnios, the difference in mean gestational age remained similar between the groups (adjusted *p* = 0.72). Additionally, the incidence of PTB was also comparable between the groups, after adjustment for the same variables (adjusted *p* = 0.59). There were no significant differences between the groups in the rates of PROM < 37 or < 34 weeks, non-PROM < 37 or < 34 weeks, or in the rate of placental abruptions after performing the same adjustment (Table 3). After controlling for the previously mentioned variables, blood group type was associated with increased rate of placental abruption among PTB < 34 weeks (*p* = 0.038), (Fig. 2). Among the 440 women who had PTB < 34 weeks, the rate of placental abruption was significantly higher in women with blood type A compared to those with blood type B (22.5% vs. 14.1%, respectively; adjusted *p* = 0.04) or O (22.5% vs. 14.0%, respectively; adjusted *p* = 0.01). The rate in group AB was 17.1%, compared to 22.5% in group A (adjusted *p* = 0.85), (Table 4).

**Table 3:** Gestational age at delivery according to ABO blood group

Demographic data	Group A (*N* = 7131)	Group B (*N* = 3787)	Group O (*N* = 7039)	Group AB (*N* = 1344)	*P*-value	*P*-value*	OR (95% CI)* B vs. A	OR (95% CI)* O vs. A	OR (95% CI)* AB vs. A
Gestational age, weeks	38.7 ± 2.0	38.7 ± 2.0	38.7 ± 1.9	38.6 ± 2.2	0.59	0.72	------	---	---
Gestational age < 37 weeks	523 (7.3)	261 (6.9)	532 (7.5)	102 (7.5)	0.68	0.59	1.02 (0.86–1.20)	1.10 (0.96–1.25)	1.06 (0.83–1.34)
Gestational age < 34 weeks	169 (2.4)	78 (2.1)	158 (2.2)	35 (2.6)	0.63	0.82	1.00 (0.75–1.34)	1.06 (0.83–1.34)	1.20 (0.81–1.78)
PROM < 37 weeks	273 (3.8)	138 (3.6)	304 (4.3)	53 (3.9)	0.30	0.21	1.00 (0.81–1.24)	1.18 (1.00-1.40)	1.04 (0.77–1.42)
PROM < 34 weeks	68 (1.0)	31 (0.8)	77 (1.1)	12 (0.9)	0.60	0.46	0.97 (0.63–1.50)	1.27 (0.90–1.78)	0.98 (0.52–1.86)
Non-PROM < 37 weeks	186 (2.6)	105 (2.8)	185 (2.6)	40 (3.0)	0.86	0.76	1.13 (0.88–1.45)	1.04 (0.84–1.28)	1.14 (0.80–1.63)
Non-PROM < 34 weeks	63 (0.9)	36 (1.0)	59 (0.8)	17 (1.3)	0.49	0.48	1.20 (0.79–1.83)	0.92 (0.71–1.47)	1.48 (0.85–1.58)
Placental abruption < 37 weeks	64 (0.9)	18 (0.5)	43 (0.6)	9 (0.7)	0.054	0.54	0.67 (0.36–1.23)	0.78 (0.49–1.23)	0.86 (0.38–1.94)
Placental abruption < 34 weeks	38 (0.5)	11 (0.3)	22 (0.3)	6 (0.4)	0.12	0.55	0.71 (0.34–1.51)	0.68 (0.38–1.23)	1.06 (0.41–2.72)

Data are mean (± SD) or N (%)

OR, odds ratio; CI, confidence interval; PROM, preterm rupture of membranes; PTB, preterm birth

*Adjusted was made for maternal age, ethnicity, multiple pregnancies, placenta previa, malformed uterus, GBS status, infectious disease, gestational hypertension and oligohydramnios

**Table 4:** Adjusted odds ratios for early preterm birth

	PROM < 34 weeks					Non-Prom < 34 weeks					Placental Abruption < 34 weeks				
				adjusted					adjusted						
**Blood type**	N (%)	OR (CI)	P	OR (CI)	P	N (%)	OR (CI)	P	OR (CI)	P	N (%)	OR (CI)	P	OR (CI)	P
A (*N* = 169)	68 (40.2)	1.00 (reference)	--	1.00 (reference)	--	63 (37.3)	1.00 (reference)	--	1.00 (reference)	---	38 (22.5)	1.00 (reference)	--	1.00 (reference)	--
B (*N* = 78)	31 (39.7)	0.98 (0.56–1.70)	0.94	0.94 (0.80–1.11)	0.48	36 (46.2)	1.44 (0.83–2.49)	0.19	1.20 (0.79–1.83)	0.40	11 (14.1)	0.57 (0.26–1.16)	0.12	0.37 (0.14–0.96)	0.04
O (*N* = 158)	77 (48.7)	1.41 (0.91–2.19)	0.12	1.14 (1.00-1.30)	0.06	59 (37.3)	1.00 (0.64–1.57)	0.99	1.02 (0.71–1.47)	0.93	22 (14.0)	0.56 (0.31–0.99)	0.046	0.37 (0.18–0.79)	0.01
AB (*N* = 35)	12 (34.3)	0.77 (0.35–1.66)	0.51	0.99 (0.78–1.25)	0.91	17 (48.6)	1.59 (0.75–3.33)	0.21	1.48 (0.85–2.58)	0.17	6 (17.1)	0.71 (0.25–1.79)	0.49	0.89 (0.28–2.81)	0.85

PROM, preterm rupture of membranes; OR, odds ratio; CI, confidence interval; P, p-value

Adjusted was made for maternal age, ethnicity, multiple pregnancies, placenta previa, malformed uterus, GBS status, infectious disease, gestational hypertension and oligohydramnios

## Discussion

In the present study, the overall rates of PTB and early PTB were 7.3% and 2.3% respectively. The rates were similar among all blood groups regardless of Rhesus factor. Additionally, there was no significant difference between the groups according to PTB etiology. In women who had early PTB, blood group A was associated with increased rate of PTB < 34 weeks due to placental abruption. This implies that women with blood group A who admit with early PTB may have an increased odds for placental abruption.

The association between maternal ABO phenotype and obstetric adverse outcomes have been suggested previously by several studies [6–14, 18–20]. Nevertheless, the data is scarce, the studies are mostly small, and the results among the different studies are inconsistent.

Franchini et al. found that, blood type O was associated with reduced risk for preeclampsia compared with non-O blood type [12]. Hiltunen et al. and Spinillo et al. reported that women with blood type AB had a 2.1- to 3.1-fold increased risk of developing preeclampsia compared to those with blood type O [6–8]. Phaloprakarn et al. reported similar results regarding maternal A and AB blood types and risk of preeclampsia [10]. Other several studies reported inconsistent association between AB blood type and the prevalence of gestational diabetes mellitus (GDM) [13, 21, 22]. Shimodaira et al. found that group AB was a risk factor for GDM in Japanese population [21], whereas Zhang et al. found that blood group AB is a protective factor for GDM [22]. A study done by our group regarding the same association found that blood type AB was associated with a lower risk of GDM when compared to other blood groups [13].

Studies on the relationship between ABO blood types and the risk of PTB are sparse and equivocal [11, 14–16]. Sameer et al., reported that only maternal-newborn pairs B-A and AB-B respectively were associated with an increased risk for PTB among 2,204 Saudi Arabian women [11]. Linn et al. [15] were unable to establish a link between a specific blood type group and PTB. In contrast, Sajan et al., reported a significant increase in the incidence of PTB and low birth wight, in blood group A compared to other blood groups, among Pakistani women [16]. In the present study rates of PTB were similar between all blood groups regardless of Rhesus factor. Nevertheless, we also found that among women with blood group A, who are admitted in preterm labor at < 34 weeks, the rate of placental abruption was higher than anticipated. This observation was not previously reported in the cited articles.

The ABO antigens are particularly expressed on the surface of red blood cells. In addition, they are also present on most epithelial and endothelial cells, T-cells, B-cells, and platelets and are detectable in most body fluids such as saliva [23]. Additionally, blood group antigens are detectable also on epithelial tissues of female reproductive organs including the uterus and ovaries [24], which may directly affect the occurrence and development of certain pregnancy outcomes [25]. Nath et al. proposed that an elevated level of pro-inflammatory cytokines up-regulate the production and activity of matrix metalloproteinases in trophoblasts, resulting in the destruction of the extracellular matrix and cell-cell interactions, which could eventually lead to the disruption of normal placental attachment [26]. The expression of these pro-inflammatory cytokines is related to blood type. Certain inflammatory cytokines, including tumor necrosis factor-alpha and soluble intercellular adhesion molecule 1 levels have been reported to be raised by genetic variants in the ABO locus SNP rs651007 especially with the A allele [27, 28]. The combination of these variations may be related with the occurrence of PTB. Additionally, Blood group A was also found to be associated with increased morbidity among preterm infants. Cakir et al., found that preterm infants with blood group A had a higher incidence of patent ductus arteriosus and bronchopulmonary dysplasia compared to infants with other blood groups. Similarly, genetic predisposition has been implicated as a probable explanation of this observation among preterm infants [25].

Although the explanation for probable relationship is still unclear, there is a possibility that the association of blood group with spontaneous PTB differs from one geographic region to another. This may explain the inconsistency between the various reports that came out from different parts of the world.

The current study is the largest study cited (more than 19,300 women) that examined the association between ABO blood types and PTB. Though ABO blood types were not found to be associated with PTB, the results also showed that types B and O appear to be protective against placental abruption in early PTB.

The present observational study did not intend to examine the mechanism behind the association found. However, the existed literature points to the existence of different expression of pro-inflammatory mediators or a particular placental factor, as in the case of preeclampsia, that may affect pregnancy outcomes. This is also supported by the fact that only placental abruption < 34 weeks was found to be related to PTB while preterm labor with intact membranes and PROM were not. Additionally, the fact that only placental abruption < 34 weeks that led to PTB was correlated, represents probably a true placental factor as compared with placental abruption near or at term.

### Study Limitations

The use of a retrospective database is the study’s principal flaw. Despite using several sources to identify exact maternal and neonatal characteristics, we were unable to collect additional information on other risk factors for PTB such as body mass index and cervical length.

Additionally, since the data was restricted to births that occurred only between 2013 and 2019, we do not have data on births that may have occurred before 2013, nor after 2019, for all women. Furthermore, we were unable to collect information regarding neonatal ABO typing in order to check for incompatibility with maternal ABO typing. Finally, the fact that this research was conducted in a single institution may restrict its generalizability. Nonetheless, a single-center study has the advantage of using similar diagnostic and management criteria. Additionally, the study is the largest among all cited articles.

## Conclusion

Blood group ABO are not associated with increased risk of PTB. A possible exception is women who have PTB < 34 weeks; among this subgroup, types B and O appear to be protective against placental abruption.

Blood type is an inexpensive and available test during the antepartum period. Hence, blood type can serve as an additional component, combined with other clinical factors, to predict the occurrence of placental abruption among women admitted in PTB < 34 weeks.
